# On the Use and Abuse of Chemical Baths

**Published:** 1857-01

**Authors:** G. Huff

**Affiliations:** Lexington, Ky.


### ARTICLE XIII.

*On the Use and Abuse of Chemical Baths.* By G. HUFF, M. D., Lexington, Ky.

WHEN we consider the deleterious effects of mercury upon the constitution at times, especially when its use has been injudiciously persevered in for some time in small, and oft repeated doses, in certain constitutional diseases in which mercury is commonly resorted to as a specific, we are led to fear that it often proves to be a greater evil than the disease itself. And if we take into view the facility and certainty of the galvanic action in the elimination of the deleterious metals from the human system, and its practical use to the community, its application must rank as one of the most valuable discoveries in modern therapeutics. I have observed, however, through life, that the more valuable any discovery to society, the greater its abuse; and in no case has this been more fully verified in the healing art within the last half century, than in the transference of metals from the human system. This branch of the profession is left entirely too much in the hands of charlatans.

Facts proving that deception has been practiced to a great extent have come within my own observation; and recently the reputed experience of the editor of the *Louisville* (daily) *Journal*, in the supposed efficacy of chemical baths, and more especially his proposed test of their action by means of ammonium, have caused great sensation in that part of the country. These circumstances led me to make an experiment with a rabbit, an animal that had never taken mercury in any form; and I herewith forward you the result, viz. a copper plate, a portion of which is nicely coated with a light metal, generally known as tin. By the mercenary, a coating like this is continually palmed off for mercury taken from the systems of those who have supposed themselves surcharged with that metal. Those persons who practice such feats of legerdemain, invariably use metallic bath tubs, the same as was done by myself in the experiment with the rabbit, and the coating of light metal upon the piece of copper is simply a deposition of tin from the tub; and the process was nothing else than electro-plating, with a rabbit in the solution.

Then again the experience of the editor referred to proves nothing, as there was no evidence of mercury having been extracted. The sulphide of ammonium, the test relied on by him, will give a black precipitate with lead, copper, bismuth, tin, and lastly, iron, provided the free acid be neutralized, which may be done in this experiment by adding excess of sulphide of ammonium, and then the black sulphide of iron will be precipitated as well as with mercury. The precipitate of mercury in a *dilute* solution turning instantaneously black, is not a characteristic of that metal, as may be tested by any person by merely putting one drop of a solution of corrosive sublimate into a tumbler full of water, and having stirred it, then adding a few drops of sulphide of ammonium, when it will be seen that the precipitate changes from a light yellow, quite rapidly to black; but unless the black sulphide be reduced, and mercury obtained from it in a metallic form, the test is not conclusive. Had a little of the supposed "black sulphide of mercury" been dried and mixed with cyanide of potassium, or carbonate of soda, and heated to redness in the sealed end of a small glass tube, the mercury, if present, would have been sublimed in metallic form in the cold portion of the tube. But it does not appear that this was done, and consequently there is no conclusive evidence that mercury was obtained from his system, but, on the contrary, he was probably deceived. The black sulphide might have been either the protosulphide of tin, or of iron, which change may take place under the following circumstances:

1st. If a patient be placed in a metallic bath tub of copper or iron, tinned, containing water with some hydrochloric acid, with a bright plate of copper under his feet, and the negative pole connected with it, and the positive pole with the bathing tub, in the course of fifteen minutes or less after the battery is in action, the copper plate will be completely coated with tin, save the portion that was covered with his feet; and if a tumbler full of the solution of the bath be tested with a few drops of sulphide of ammonium, it will give a black precipitate of protosulphide of tin; which it would not have done previous to the battery having been put in action.

2d. The same effect will be produced if the patient has the negative pole in his hand, with his feet on a polished plate, *it being insulated*, and the positive pole in contact with the bathing tub. The person in connection with the negative pole merely serves as an electrode to the plate on which a deposition of metal (tin) is wanted for deception. This experiment may be made very readily by any person having a battery of sufficient power. Persons in connection with a battery, are in this way led to believe that the metal thus deposited upon the plate beneath their feet, passed from their system, as they felt during the process (of electro-plating,) as if they were "pierced with ten thousand needles." This would answer a very good purpose if such persons would recover from their infirmities in consequence of their belief. But, alas for the poor dupes! they remain without benefit. I am acquainted with a person that has reaped an abundant harvest within the last seven months by such duplicity. And I fear, as a general thing, the profession is not as well posted in *electro-chemistry*, as they should be; as I have known some physicians to witness the *modus operandi*, as aforesaid, and supposed the deposit of tin upon copper was the "Simon pure" from the human system.

3d. If a zinc bathing tub be used under the same circumstances as the preceding, the same effect upon a polished plate will follow, and the solution will give a blackish precipitate, which is owing to the iron always being present in the commercial zinc, which latter, when pure, gives from its neutral solution a white precipitate. It is always necessary to add the sulphide of ammonium in slight excess, to neutralize the acid of the bath, as iron will not precipitate in acid solutions. If there is much organic matter present in an acid bath, the sulphide of ammonium will give a dirty sulphur precipitate.

It is certain that very few persons in any community are aware that tin can be eliminated in solution from a bath tub, and deposited upon a plate of copper within the said tub; hence the credulity of the public is taxed by those who are greedy for gain. In order to manage fairly and effectually, those persons who suppose themselves surcharged with mercury, all metallic bath tubs should be dispensed with, and those only should be used which are made of non-conducting material, such as porcelain, stone, glass or marble. A simple porcelain foot tub, is as good utensil as can be used for the purpose, as it is not at all necessary to immerse the whole person; the immersion of the feet in only a few inches of the solution being all that is required for the process of transferring metals from the human system.

It is truly unfortunate that the medical profession should be so prejudiced against other modes of treating diseases than such as they learned in early life, just as if science is not progressive. If such prejudices did not exist, the public would not suffer so much by empiricism ; and if they patronize men without science, it is certain that they have lost confidence in legitimate practice.—*N. Y. Medical Times.*

